# Health-related quality of life in young adults born small for gestational age: a prospective cohort study

**DOI:** 10.1186/s12955-022-01948-4

**Published:** 2022-03-24

**Authors:** Cathrin Vano Mehl, Ingrid Marie Husby Hollund, Johanne Marie Iversen, Stian Lydersen, Paul Jarle Mork, Eero Kajantie, Kari Anne I. Evensen

**Affiliations:** 1 Department of Clinical and Molecular Medicine, Norwegian University of Science and Technology, NO-7491 Trondheim, Norway; 2 Department of Physical Medicine and Rehabilitation, St. Olavs Hospital, Trondheim University Hospital, Trondheim, Norway; 3 Department of Internal Medicine, Nordland Hospital Trust, Bodø, Norway; 4 Department of Clinical Medicine, UiT Arctic University of Norway, Tromsø, Norway; 5 Department of Mental Health, Norwegian University of Science and Technology, Trondheim, Norway; 6 Department of Public Health and Nursing, Norwegian University of Science and Technology, Trondheim, Norway; 7 Public Health Promotion Unit, Finnish Institute for Health and Welfare, Helsinki, Oulu, Finland; 8 PEDEGO Research Unit, MRC Oulu, Oulu University Hospital and University of Oulu, Oulu, Finland; 9 Children’s Hospital, Helsinki University Hospital and University of Helsinki, Helsinki, Finland; 10 Unit for Physiotherapy, Trondheim Municipality, Trondheim, Norway; 11 Children’s Clinic, St. Olavs Hospital, Trondheim University Hospital, Trondheim, Norway; 12 Department of Physiotherapy, Oslo Metropolitan University, Oslo, Norway

**Keywords:** Small for gestational age, Health-related quality of life, SF-36, Self-perceived health status, Long-term outcome, Young adulthood, Longitudinal

## Abstract

**Background:**

Individuals born small for gestational age (SGA) have an increased risk of several adverse health outcomes, but their health-related quality of life (HRQoL) across young adulthood has yet to be studied. The main aim of this study was to investigate if being born SGA at term is associated with poor HRQoL at 32 years of age. A second aim was to explore longitudinal changes in HRQoL from age 20 to 32 years.

**Methods:**

In the prospective NTNU Low Birth Weight in a Lifetime Perspective study, 56 participants born SGA and 68 non-SGA control participants completed the Short Form 36 Health Survey (SF-36) at age 32 years to assess HRQoL. The SF-36 was also administrated at age 20 and 28 years. Longitudinal changes in the eight SF-36 domains and the two component summaries from 20 to 32 years were analyzed by linear mixed models. In total, 82 adults born SGA and 98 controls participated at least once and were included in the longitudinal analyses.

**Results:**

At age 32 years the participants born SGA scored 14.8 (95% CI 4.7 to 25.3) points lower in the SF-36 role-physical domain compared with the control group, i.e. more problems with work or other daily activities due to physical health problems. The longitudinal analyses showed significant group differences from 20 to 32 years in the role-emotional domain, and in the physical and mental component summaries. Among participants born SGA, the physical component summary decreased from age 20 to 28 years (-3.2, 95% CI -5.0 to -1.8), while the mental component summary (6.0, 95% CI 2.9 to 8.6) and role-emotional domain score (19.3, 95% CI 9.9 to 30.3) increased, but there were no further changes from 28 to 32 years. There were no longitudinal changes in the control group from 20 to 32 years.

**Conclusion:**

Overall, individuals born SGA at term reported similar HRQoL at age 32 years compared with non-SGA controls. Self-perceived mental health improved during young adulthood among individuals born SGA, while self-perceived physical health deteriorated. The latter findings warrant further investigation.

**Supplementary Information:**

The online version contains supplementary material available at 10.1186/s12955-022-01948-4.

## Background

Being born small for gestational age (SGA; birth weight < 10th percentile for gestational age) at term involves an increased risk of adverse health outcomes throughout life [1]. As infants, those born SGA have higher morbidity and mortality than other term newborns [2]. A recent meta-analysis reported that children born SGA score on average 0.23 SD lower on cognitive tests than their peers born appropriate for gestational age. This difference corresponds to approximately 3.5 IQ points [3]. Accordingly, being born SGA may increase the risk of learning difficulties [4], lower academic performance [5–7], and enrollment in special education [6, 8]. Furthermore, children and adolescents born SGA at term are more likely to report attentional difficulties [4, 9] and symptoms of anxiety and depression [10]. These challenges resemble those that have been described in individuals born preterm, but outcomes of individuals born SGA have been less studied.

Some studies also indicate that consequences of being born SGA extend into adulthood, including increased risk of cardiovascular disease, obesity and type 2 diabetes mellitus [1]. Additionally, a population-based registry study reported increased risk of hospitalization for mental disorders among adolescents and young adults born SGA [11]. These findings are in line with results from our population-based cohort study, where longitudinal analyses showed that individuals born SGA at term had a striking increase in psychiatric morbidity during the transition into adulthood [12].

Health-related quality of life (HRQoL) can be defined as an individual’s or group’s perceived physical and mental health over time [13]. Despite the well-documented health consequences of being born SGA, studies investigating HRQoL in people born SGA at term are sparse. According to a follow-up of the UK 1970 birth cohort at 26 years, there were no long-term emotional or social consequences of being born SGA (birth weight < 5th percentile), and SGA was not associated with lower life satisfaction [6]. Another study reported similar HRQoL at 50 years of age in a group with birth weight < 10th percentile and a group with birth weight ≥ 10th percentile [14]. In contrast, results from another follow-up study at 50 years of age suggest that both low and high-range birth weights increase the risk of low QoL and low satisfaction with life [15]. A recent systematic review suggests that children and adults with short stature, which is one of the most common complications of SGA birth weight [1], may experience poorer quality of life [16]. There are no studies examining HRQoL longitudinally in adults born SGA at term. We have previously found that 20-year-olds born SGA (birth weight < 10th percentile) perceived their mental health and social functioning as poorer and reported more role limitations due to emotional problems than controls [17]. We have now reassessed this cohort across young adulthood, allowing for longitudinal analyses of their HRQoL.

The main aim of this study was to investigate if being born SGA at term is associated with poor HRQoL at 32 years of age. A second aim was to explore longitudinal changes in HRQoL from age 20 to 32 years.

## Methods

### Study design and study population

This prospective cohort study is part of the NTNU Low Birth Weight in a Lifetime Perspective study. Two groups of adults born at term (gestational age ≥ 37 weeks) in 1986-1988 were examined at 20, 28 and 32 years of age. One group was born SGA (birth weight < 10th percentile) and one group was born non-SGA (birth weight ≥ 10th percentile). Participants attended study visits at several time points from birth and up to 32 years of age. HRQoL was examined as part of a larger data collection, including anthropometric measurements, examination of lung function, physical fitness, motor function and visual function. Individuals who were unable to meet for clinical examination were invited to answer questionnaires only. Data for the follow-up at age 32 years were collected from September 2019 through March 2021.

**Fig. 1:**
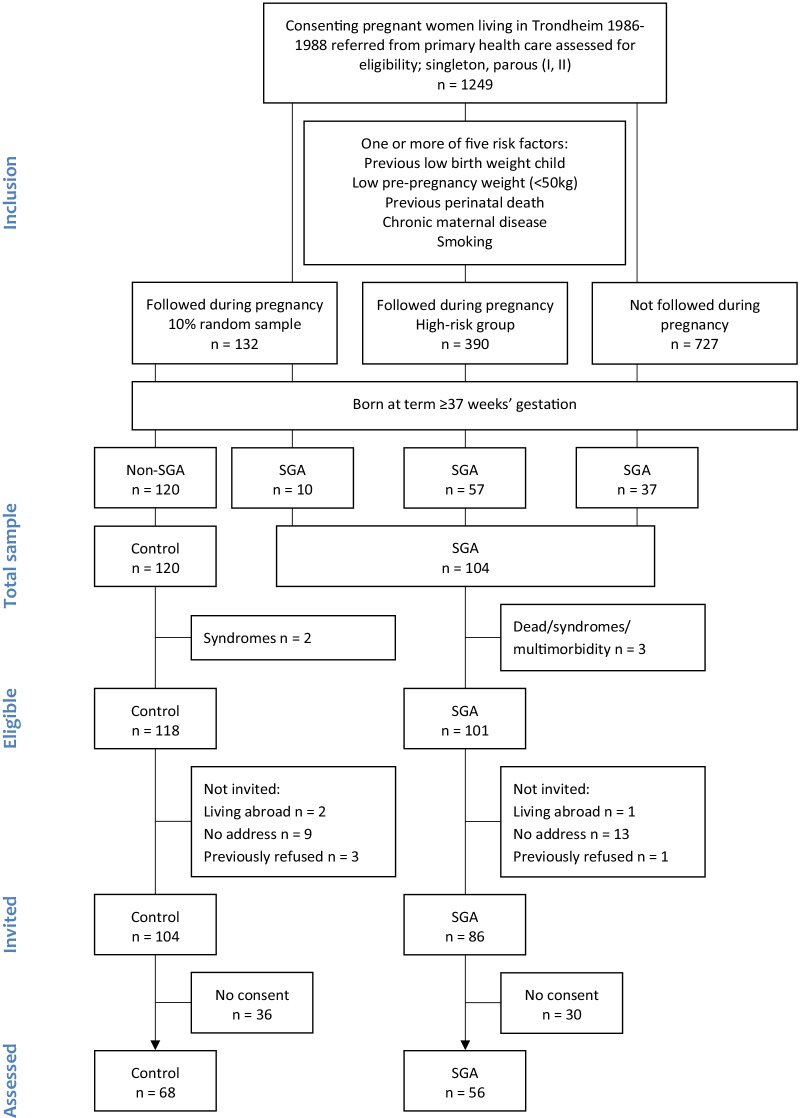
Flow of participants at 32 years. SGA, small for gestational age

The participants were initially included as part of a multicenter study that recruited pregnant women before gestational week 20 [18, 19]. They were eligible if they were carrying a singleton and had been pregnant one or two times before. In the region of Trondheim, Norway, 1249 women consented to participate. Using a sealed envelope method, a 10% random sample (n = 132) was selected for follow-up, representative of the pregnant population at the study site. Women at high risk of delivering an SGA infant were selected for follow-up if they fulfilled one or more defined risk criteria; a previous perinatal death or child with low birth weight, cigarette smoking at conception, pre-pregnancy weight < 50 kg, or chronic diseases (hypertension, renal or heart disease) (n = 390). Women in the random sample (n = 132) and in the high-risk group (n = 390) were followed through pregnancy and their babies were examined at birth. The remaining women were not selected for detailed follow-up (n = 727). All infants born SGA at term to mothers in either group were included in the SGA group (n = 104). The control group (n = 120) comprised all infants born non-SGA from the random sample only. Flow of the participants that were included at the 32-year follow-up is illustrated in Fig. 1.

### SGA participants

The SGA group included 104 individuals born at term with a birth weight < 10th percentile for gestational age, corrected for sex and parity, according to a reference standard using data from the Norwegian Medical Birth Registry [18]. Gestational age was determined based on the first day of the mother’s last menstrual period (LMP) when this was recalled accurately ± 3 days. An ultrasound estimate was used if the LMP-based gestational age was not recalled or differed by more than 14 days from the ultrasound-based gestational age. Individuals with multimorbidity, congenital syndromes or who died before follow-up were excluded (n = 3). At the 32-year follow-up, 15 individuals born SGA were living abroad, could not be reached, or had previously refused to participate. Out of 86 invited subjects, 30 did not consent, leaving 56 (25 males, 31 females) participants born SGA (65% of 86 invited and 55% of 101 eligible) (Fig. 1). HRQoL was assessed for 66 (34 males, 32 females) and 55 (24 males, 31 females) SGA participants at age 28 and 20 years, respectively. In total, 82 adults born SGA participated at least once and were included in the longitudinal analyses.

### Control participants

The control group included 120 individuals born at term with a birth weight ≥ 10th percentile. Two were excluded at birth due to congenital syndromes. At the 32-year follow-up, 14 controls were living abroad, could not be reached, or had previously refused to participate. Out of 104 invited subjects, 36 did not consent, leaving 68 (29 males, 39 females) participants in the control group (65% of 104 invited and 58% of 118 eligible) (Fig. 1). HRQoL was assessed for 86 (38 males, 48 females) and 74 (31 males, 43 females) control participants at age 28 and 20 years, respectively. In total, 98 controls participated at least once and were included in the longitudinal analyses.

### Non-participants

There were no substantial differences between participants and those who did not consent to participate at 32 years regarding gestational age, birth weight, birth length, head circumference, ponderal index, maternal age at delivery, parental socioeconomic status (SES) or sex in either group (Additional file 1: Table A1).

### Background characteristics

Gestational age, birth weight, birth length, head circumference, sex and maternal age were recorded at birth. Ponderal index (g/cm^3^) was calculated based on birth weight and length. Parental SES was calculated according to Hollingshead’s Two Factor Index of Social Position [20], based on a combination of the parents’ education and occupation recorded at the 14-year follow-up and supplemented at the 19-year follow-up. The social class was rated from 1 (lowest) to 5 (highest).

At the follow-up at 32 years of age, the participants’ height and weight were measured. Height was measured to the nearest mm. Weight was measured by bioelectric impedance analysis using a Seca medical Body Composition Analyzer (Seca® mBCA 515) with a 100 g accuracy. Body mass index (BMI, kg/m^2^) was calculated. Ten SGA participants and seven control participants could not meet at the clinical examination, and data on height and weight were collected by self-report.

The highest level of education was assessed by self-report at the 32-year follow up. We classified the highest completed level of education according to International Standard Classification of Education (ISCED) levels 1 through 8 and defined three categories. Lower secondary education or lower (ISCED levels 1-2) refers to no more than 10 years of education. Intermediate education (ISCED levels 3-5) refers to 11-14 years of education, but not higher education. Lower tertiary education or higher (ISCED levels 6-8) refers to a bachelor’s degree or higher.

### Health-related quality of life: Short Form 36 Health Survey (SF-36)

The Short Form 36 Health Survey (SF-36) was used to assess HRQoL. The SF-36 is a multi-purpose generic health questionnaire, that consists of 36 statements measuring eight health concepts: (1) physical functioning; (2) role limitations due to physical health problems (role-physical); (3) bodily pain; (4) general health perceptions; (5) vitality; (6) social functioning; (7) role limitations due to emotional problems (role-emotional); and (8) general mental health perceptions. The SF-36 was designed to examine health status, and the construction allows for use in research, health policy evaluations, clinical practice and general population surveys [21]. The Norwegian version of SF-36 has been evaluated in a Norwegian population of patients, and was found to have acceptable reliability and validity [22].

The SF-36 provides insight into the individual’s understanding of their own health and gives information about well-being and ability to perform everyday tasks. The participants answer the questions by marking the option that suits them best. Response alternatives are dichotomized for the role-emotional and role-physical domains, while the remaining domains have three to six alternatives on an ordinal scale. Raw item scores are coded, summed and transformed into an aggregate score for each of the eight domains, ranging from 0 to 100% [23, 24]. Higher scores indicate higher level of functioning and favorable health outcomes. The eight domains are aggregated into two summary measures, the physical component summary and the mental component summary. The component summaries are given as T-scores, based on an average of 50 points and a standard deviation (SD) of 10 points. The physical component summary has contributions mainly from the domains physical functioning, role-physical and bodily pain, while the domains social functioning, role-emotional and mental health contribute mainly to the mental component summary. Both component summaries correlate with the domains vitality, general health and social functioning [24].

### Ethical approval and consent

The Regional Committee for Medical and Health Research Ethics in Central Norway (23879) approved the study. All participants gave written informed consent to participate in the project. None of the examinations were painful or harmful. Participants were given feedback on the examinations, and if necessary, referred to appropriate health services. Participants were offered a compensation of NOK 500 (about 50 Euros) in addition to coverage of travel expenses.

### Statistical analysis

Background characteristics of the SGA and control participants were compared using Student’s *t*-test for continuous data, Exact Mann-Whitney U test for ordinal data and Pearson’s Chi square test for dichotomous variables.

Group differences in SF-36 domains and component summaries were analyzed using linear regression, adjusted for sex. Missing items were handled according to the manual for SF-36 [23], using mean imputation on a scale if at least 50% of the items on the scale had available data. The correlation between adult height and SF-36 variables was assessed using Spearman’s rank correlation coefficient (r_s_). Estimated changes in domains and component summaries from 20 to 32 years were analyzed using linear mixed models. SF-36 scores were entered separately as dependent variables, time and group and their interaction as fixed factors, sex as fixed factor, and participant as random factor. Normality of residuals was judged by visual inspection of Q-Q plots. Due to some deviations from normality, we used bootstrapping with B = 2000 bootstrap samples and bias corrected and accelerated (BC_a_) method. Ninety-five percent confidence intervals (CI) are reported where relevant, and a two-sided *p*-value < 0.05 was considered statistically significant. SPSS 26.0 was used for data analyses.

## Results

### Background characteristics

Table 1 shows background characteristics of the participants born SGA at term and controls at the 32-year follow-up. As expected, the SGA group was smaller at birth than the control group, measured by weight, length, head circumference and ponderal index. The mothers of the participants born SGA were younger at delivery (*p* = 0.001). At the 32-year follow-up, those who were born SGA were shorter than the control group, but there were no significant correlations between adult height and SF-36 scores (r_s_ <  ± 0.200, *p* > 0.150). There were no group differences in weight, BMI, parental SES, educational attainment, sex, or age. Mean age at the previous follow-ups were 19.8 (0.7) and 28.6 (0.5) in the SGA group, and 19.7 (0.6) and 28.5 (0.4) in the control group.

**Table 1:** Background characteristics of participants born SGA at term and controls

	SGA (n = 56)		Control (n = 68)		
	Mean	(SD)	Mean	(SD)	*p*-value
Gestational age (weeks)	39.7	(1.2)	39.8	(1.2)	0.454
Birth weight (g)	2916	(205)	3695	(459)	< 0.001
Birth length (cm)^a^	48.6	(2.0)	51.2	(1.9)	< 0.001
Birth head circumference (cm)^b^	33.9	(1.1)	35.4	(1.2)	< 0.001
Ponderal index^a^ (g/cm^3^)	2.6	(0.2)	2.8	(0.3)	< 0.001
Maternal age at delivery (years)	28.2	(3.3)	30.5	(4.3)	0.001
Parental SES (1-5)^c^	3.5	(1.2)	3.7	(1.1)	0.442
Age at current follow-up (years)	32.5	(0.6)	32.6	(0.5)	0.485
Height (cm)	169.5	(9.2)	174.5	(10.0)	0.005
Weight (kg)	72.6	(17.0)	76.1	(15.4)	0.232
BMI (kg/m^2^)	25.1	(4.9)	24.9	(4.3)	0.815

	n	(%)	n	(%)	
Female	31	(55)	39	(57)	0.823
Education at follow-up					
Lower secondary or lower (ISCED 1-2)	2	(4)	0	(0)	
Intermediate (ISCED 3-5)	22	(39)	23	(34)	0.214
Lower tertiary or higher (ISCED 6-8)	32	(57)	45	(66)	

BMI, body mass index; ISCED, International Standard Classification of Education; SD, standard deviation; SES, socioeconomic status (1-5, where 5 is highest); SGA, small for gestational age

^a^Data missing for seven SGA participants and four control participants

^b^Data missing for six SGA participants and five control participants

^c^Data missing for nine SGA participants and eleven control participants

### Health-related quality of life at 32 years

SF-36 domain scores and component summaries at 32 years are presented in Table 2. The SGA group scored 14.8 (95% CI 4.7 to 25.3, *p* = 0.009) points lower than the control group in the role-physical domain, corresponding to 0.75 times the SD among the controls. None of the other domains or component summaries differed significantly between the groups.

**Table 2:** Health-related quality of life in participants born SGA at term and controls at 32 years

	SGA (n = 56)		Control (n = 68)				
	Mean	(SD)	Mean	(SD)	Mean difference (95% CI)^a^		*p*-value
*Domains*							
Physical functioning	93.7	(13.4)	96.3	(7.2)	-2.7	(-6.6 to 0.6)	0.211
Role-physical^b^	78.2	(37.3)	92.6	(19.8)	-14.8	(-25.3 to -4.7)	0.009
Bodily pain	75.0	(26.3)	78.2	(20.5)	-3.3	(-11.9 to 4.8)	0.435
General health	78.0	(22.1)	78.3	(18.5)	-0.4	(-7.8 to 6.6)	0.917
Vitality^c^	52.4	(18.9)	55.1	(18.4)	-2.8	(-9.5 to 3.8)	0.419
Social functioning	87.3	(22.8)	89.9	(19.7)	-2.7	(-10.8 to 4.9)	0.492
Role-emotional	87.5	(29.5)	92.2	(23.1)	-4.7	(-14.1 to 4.5)	0.336
Mental health^b^	76.0	(15.2)	79.4	(14.1)	-3.4	(-8.4 to 1.6)	0.212
*Component summaries*							
Physical component summary^bc^	53.0	(9.6)	55.2	(5.5)	-2.2	(-5.2 to 0.6)	0.134
Mental component summary^bc^	49.3	(9.4)	50.6	(9.6)	-1.3	(-4.7 to 2.1)	0.463

Domain scores are given in percentage (range 0-100) and higher scores indicate better health-related quality of life

Component summaries are given as T-scores based on an average of 50 points and a standard deviation of 10 points

CI, confidence interval; SD, standard deviation; SGA, small for gestational age

^a^Mean difference adjusted for sex, confidence interval and *p*-value based on bias-corrected and accelerated bootstrap (BCa)

^b^Data missing for one SGA participant

^c^Data missing for one control participant

### Changes in health-related quality of life from 20 to 32 years

**Fig. 2:**
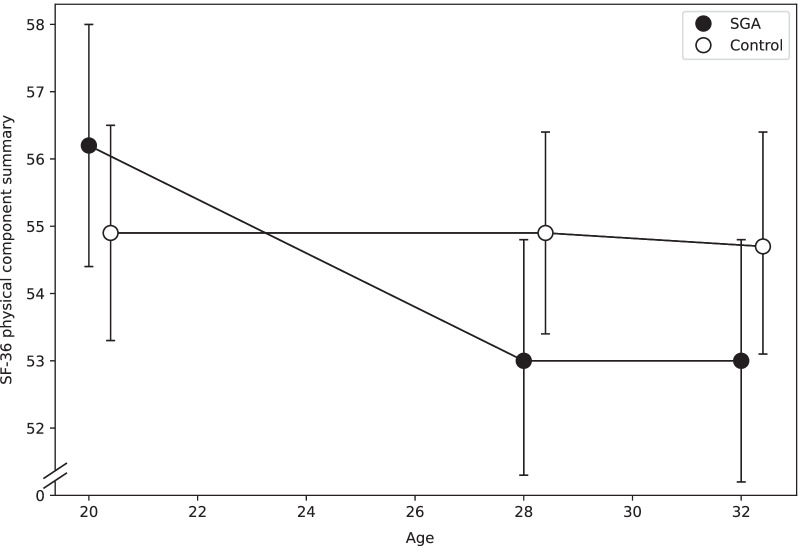
Physical component summaries with 95% confidence interval at 20, 28 and 32 years of age. T-scores are given. A higher score indicates better physical health-related quality of life. SF-36, Short Form 36 Health Survey; SGA, small for gestational age

**Fig. 3:**
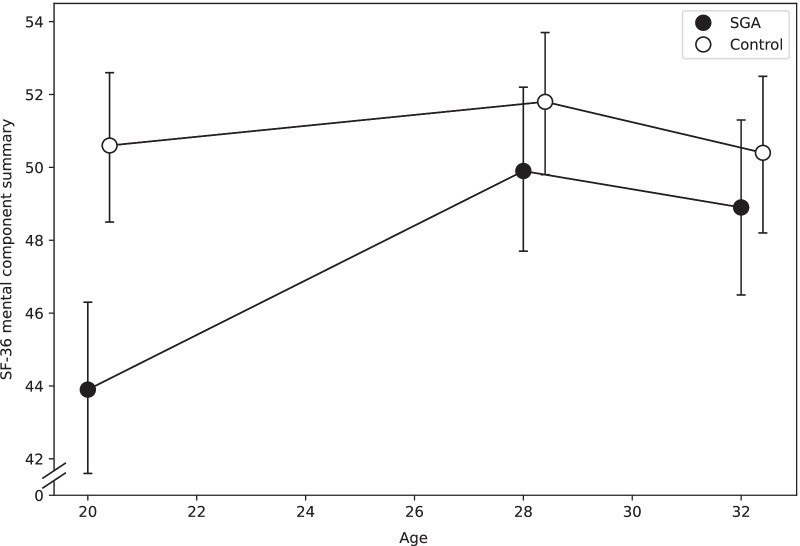
Mental component summaries with 95% confidence interval at 20, 28 and 32 years of age. T-scores are given. A higher score indicates better mental health-related quality of life. SF-36, Short Form 36 Health Survey; SGA, small for gestational age

Longitudinal changes in SF-36 domains and component summaries from 20 to 32 years are presented in Table 3, and Figs. 2 and 3. Results from the previous follow-ups at 20 and 28 years are shown in additional files (Additional file 2, 3: Tables A2 and A3).

**Table 3:** Estimated changes in health-related quality of life from 20 to 32 years

	SGA (n = 82)						Control (n = 98)						
	20 to 28 years			28 to 32 years			20 to 28 years			28 to 32 years			
	B	(95% CI)	*p*-value	B	(95% CI)	*p*-value	B	(95% CI)	*p*-value	B	(95% CI)	*p*-value	*p*-value^a^
*Domains*													
Physical functioning	-1.9	(-4.1 to 0.1)	0.141	-0.5	(-3.2 to 2.3)	0.767	0.9	(-0.8 to 2.2)	0.359	-0.7	(-2.2 to 0.6)	0.372	0.269
Role-physical	-3.7	(-10.8 to 0.8)	0.391	-1.8	(-10.9 to 8.8)	0.701	-1.6	(-6.2 to 2.9)	0.552	2.4	(-2.9 to 7.3)	0.388	0.599
Bodily pain	-6.0	(-10.7 to -2.9)	0.053	0.1	(-5.8 to 6.3)	0.967	0.4	(-4.7 to 5.7)	0.870	-1.9	(-5.9 to 1.5)	0.379	0.302
General health	4.2	(-0.3 to 9.0)	0.084	-2.0	(-6.6 to 2.4)	0.317	3.9	(0.2 to 7.8)	0.034	-4.8	(-7.4 to -3.2)	0.006	0.611
Vitality	2.9	(-2.3 to 7.2)	0.323	-2.8	(-8.5 to 3.1)	0.309	0.6	(-2.8 to 4.2)	0.712	-2.0	(-5.0 to 0.6)	0.229	0.804
Social functioning	3.3	(-1.9 to 8.2)	0.251	1.1	(-3.7 to 5.7)	0.690	1.5	(-1.2 to 4.0)	0.298	-3.8	(-6.9 to -0.4)	0.020	0.146
Role-emotional	19.3	(9.9 to 30.3)	0.001	-1.4	(-8.9 to 5.9)	0.719	3.1	(-0.4 to 6.4)	0.117	-1.6	(-5.1 to 2.0)	0.390	0.003
Mental health	7.6	(3.9 to 11.4)	0.001	-2.8	(-6.6 to 1.2)	0.152	2.3	(-0.03 to 4.8)	0.052	-1.8	(-4.1 to 0.2)	0.160	0.111
*Component summaries*													
Physical component summary	-3.2	(-5.0 to -1.8)	0.004	-0.1	(-2.3 to 2.4)	0.923	0.003	(-1.3 to 1.3)	0.998	-0.1	(-1.2 to 0.7)	0.856	0.030
Mental component summary	6.0	(2.9 to 8.6)	0.001	-1.0	(-3.5 to 1.6)	0.418	1.2	(-0.1 to 2.6)	0.091	-1.4	(-2.8 to 0.2)	0.051	0.006

Regression coefficient B and bias-corrected and accelerated bootstrap (BC_a_) confidence interval (CI) in mixed model analyses with SF-36 scores as dependent variables, time and group and their interaction as fixed factors, adjusted for sex

SGA, small for gestational age

^a^*p*-value for group differences in estimated longitudinal changes (interaction group*time) from 20 to 32 years

There were significant group differences from 20 to 32 years in role-emotional (Table 3), as well as in the physical and mental component summaries (Figs. 2, 3). From 20 to 28 years, the scores in role-emotional, mental health and the mental component summary increased in the SGA group, whereas the physical component summary decreased, but there were no further changes from 28 to 32 years (Table 3). In the control group, scores in general health increased from 20 to 28 years and decreased from 28 to 32 years, while scores in social functioning decreased from 28 to 32 years.

## Discussion

Overall, we found similar HRQoL at 32 years of age among individuals born SGA at term and non-SGA controls, except that the participants born SGA reported more role limitations due to physical health problems. The longitudinal analyses showed that individuals born SGA improved their mental component summary across young adulthood, while their physical component summary decreased. They also reported less role limitations due to emotional problems. These changes were seen from age 20 to 28 years; there were few changes in mean scores from 28 to 32 years. In the control group, the corresponding scores remained stable from age 20 to 32 years.

Strengths of this study include the prospective design, the longitudinal data during adulthood and the use of a valid and reliable questionnaire to assess HRQoL [25]. However, loss to follow-up is a challenge in any long-term study. The small sample size may reduce the power to detect differences between the groups and limit the generalizability of the study. Findings of no difference should therefore be interpreted with caution. In all, 65% of the invited in both groups participated at the 32-year follow-up, and bias due to loss to follow-up is unlikely, as there were no differences in background characteristics between participants and those who did not consent to participate. Thus, we can assume that our participants were representative of the initial sample. The linear mixed models include all participants in the longitudinal analyses, also those with missing data at some time point. The results are unbiased under the missing at random assumption, and do not rely on the more restrictive missing completely at random assumption. The longitudinal data allowed for assessment of changes in self-perceived health status across young adulthood. Although the number of comparisons is quite large, the role of chance is reduced by calculating the component summaries, which are aggregates of the eight domains.

There are several definitions of HRQoL [26]. The SF-36 provides a broad perspective on the understanding of this complex and multidimensional concept. It measures self-perceived health status across eight domains and allows an insight into the participants’ well-being from their own point of view. A subjective assessment of functioning level and health may be more relevant than objective evaluations since personal expectations and values are incorporated. Although self-reports can be affected by cognitive ability and are prone to social desirability bias, this may be the most optimal strategy, as HRQoL emphasizes the individual’s subjective perspective.

The 10th percentile definition of SGA does not necessarily identify infants who experienced intrauterine growth restriction (IUGR). The group comprises pathologically small babies, in addition to genetically small babies who are otherwise healthy and not growth restricted. Other infants with IUGR may have been classified as non-SGA due to a birth weight above the cut off value. This misclassification may dilute the real effect of IUGR and could contribute to smaller differences between the groups in this study.

Adult height differed between the groups. Although others have reported an association between short stature and poor QoL [16], we did not find that adult height was correlated to SF-36 scores.

Studies of HRQoL in term-born SGA populations are sparse. We found overall similar SF-36 scores among participants born SGA and non-SGA controls at age 32 years, consistent with the following studies [6, 14]. Strauss et al. found that 26-year-olds born SGA at term were as satisfied with life as those born with normal birth weight, even though the definition of SGA was stricter than ours (birth weight < 5th percentile) [6]. Spence et al. compared 50-year-olds born with birth weight < 10th percentile to those with birth weight ≥ 10th percentile, and found no significant difference in HRQoL, measured by SF-36 [14]. In the current study, individuals born SGA scored 14.8 points, or 0.75 SD, lower than controls in the role-physical domain at age 32 years, which may be interpreted as problems with daily activities, such as work, due to physical health problems [21]. The reason for this discrepancy with the study of Spence et al. is not evident, although one may speculate that our younger 32-year-old participants experience physical problems as a larger role limitation than do older individuals. Furthermore, individuals born SGA had a decrease of 3.2 points in the physical component summary score from age 20 to 28 years, and the overall change from age 20 to 32 years differed significantly from the control group. One study has estimated the minimal clinically important difference for the SF-36 component summaries to be approximately 4 points [27]. Although our difference was slightly smaller, there may be reason to believe that the change in self-perceived physical health has an impact on the participants’ daily life. Impaired fetal growth has been linked to factors that could influence a person’s self-perceived physical health, such as ischemic heart disease [28], an unfavorable metabolic profile [29], type 2 diabetes [30] and hypertension [31]. Additionally, results from two Swedish registry studies suggest that low birth weight is associated with reduced cardiorespiratory fitness and grip strength in young adulthood [32, 33]. However, physical activity has yet to be studied in term-born SGA populations.

Our findings of improved scores in the role-emotional domain and in the mental component summary by 6.0 points from age 20 to 28 years suggest a substantial positive impact on the lives of adults born SGA in relation to the minimal clinically important difference of 4 points. Although there are no other studies of longitudinal changes in HRQoL in term-born SGA populations, our findings of improved self-perceived mental health are in contrast to our previous findings of increased psychiatric morbidity in SGA individuals between 14 and 20 years, and then further to 26 years of age [12]. Possible explanations for this discrepancy may involve differences in methodology and age at examination. A psychiatric diagnosis is based on a semi-structured interview allowing the clinician to objectively evaluate symptoms and level of functioning according to diagnostic criteria. Self-perceived mental health is a subjective evaluation based on the individual’s own goals and standards within the current setting. Thus, the individual may perceive their situation differently than a clinician. Also, as increased psychiatric morbidity was found at 26 years, we could speculate that potential challenges in the transition to adulthood have stabilized between 26 and 28 years. Nevertheless, it is reassuring that both the physical and the mental component summary have remained stable from age 28 to 32 years.

This study is the first to explore longitudinal changes of HRQoL across young adulthood among individuals born SGA at term. The magnitude of the improved self-perceived mental HRQoL among participants born SGA from 20 to 28 years could make a difference in their everyday life and their ability to perform daily tasks. However, the longitudinal development of their self-perceived physical HRQoL calls for further investigation of their physical health and fitness. Those who are born SGA at term represent a substantial number of affected individuals, and their potential health deficits could have a noticeable impact on public health.

## Conclusions

Overall, individuals born SGA at term reported similar HRQoL at age 32 years compared with non-SGA controls. Self-perceived mental health improved during young adulthood among individuals born SGA, while self-perceived physical health deteriorated. The latter findings warrant further investigation.

## Supplementary Information

**Additional file 1: Table A1** Background characteristics of participants and those who did not consent to participate at 32 years

**Additional file 2: Table A2** Health-related quality of life in participants born SGA at term and controls at 20 years

**Additional file 3: Table A3** Health-related quality of life in participants born SGA at term and controls at 28 years

## Data Availability

The datasets generated and/or analyzed during the current study are not publicly available because permission has not been applied for from neither the participants nor the Ethical Committee but are available from the corresponding author on reasonable request.

## Abbreviations

BCa: Bias-corrected and accelerated bootstrap
BMI: Body mass index
CI: Confidence interval
HRQoL: Health-related quality of life
ISCED: International Standard Classification of Education
IUGR: Intrauterine growth restriction
LMP: Last menstrual period
SD: Standard deviation
SES: Socioeconomic status
SF-36: Short Form 36 Health Survey
SGA: Small for gestational age
