# Supplementary material for: Health-related quality of life in young adults born small for gestational age: a prospective cohort study
Source: Health Qual Life Outcomes. 2022 Mar 24;20:49. doi: 10.1186/s12955-022-01948-4 (PMC8944049; doi:10.1186/s12955-022-01948-4)
Supplement: Supplementary file 1 — Additional file 1: Table A1 Background characteristics of participants and those who did not consent to participate at 32 years [file 12955_2022_1948_MOESM1_ESM.docx]

**Table A1** Background characteristics of participants and those who did not consent to participate at 32 years

|  | **SGA** | | | |  |  | **Control** | | | |  |
| --- | --- | --- | --- | --- | --- | --- | --- | --- | --- | --- | --- |
|  | **Participants (n = 56)** | | **Non-participants (n = 30)** | |  |  | **Participants (n = 68)** | | **Non-participants (n = 36)** | |  |
|  | **Mean** | **(SD)** | **Mean** | **(SD)** | ***p*-value** |  | **Mean** | **(SD)** | **Mean** | **(SD)** | ***p*-value** |
| Gestational age (weeks) | 39.7 | (1.2) | 39.4 | (1.2) | 0.301 |  | 39.8 | (1.2) | 39.4 | (1.3) | 0.095 |
| Birth weight (g) | 2916 | (205) | 2923 | (227) | 0.885 |  | 3695 | (459) | 3703 | (430) | 0.930 |
| Birth length (cm)^a^ | 48.6 | (2.0) | 48.2 | (1.3) | 0.494 |  | 51.2 | (1.9) | 50.8 | (1.6) | 0.410 |
| Birth head circumference (cm)^b^ | 33.9 | (1.1) | 34.0 | (1.1) | 0.763 |  | 35.4 | (1.2) | 35.4 | (1.1) | 0.894 |
| Ponderal index^a^ | 2.6 | (0.2) | 2.6 | (0.2) | 0.831 |  | 2.8 | (0.3) | 2.8 | (0.2) | 0.374 |
| Maternal age at delivery (years)^c^ | 28.2 | (3.3) | 29.4 | (4.4) | 0.194 |  | 30.5 | (4.3) | 29.2 | (4.0) | 0.133 |
| Parental SES (1-5)^d^ | 3.5 | (1.2) | 3.3 | (1.6) | 0.806 |  | 3.7 | (1.1) | 3.9 | (1.0) | 0.666 |
|  | **n** | **(%)** | **n** | **(%)** |  |  | **n** | **(%)** | **n** | **(%)** |  |
| Female | 31 | (55) | 13 | (43) | 0.288 |  | 39 | (57) | 17 | (47) | 0.324 |

SD, standard deviation; SES, socioeconomic status (1-5, where 5 is highest); SGA, small for gestational age

^a^ Data missing for seven SGA participants, seven SGA non-participants, five control participants and one control non-participant

^b^ Data missing for six SGA participants, eight SGA non-participants, five control participants and one control non-participant

^c^ Data missing for one SGA non-participant

^d^ Data missing for nine SGA participants, eighteen SGA non-participants, eleven control participants and fifteen control non-participants
