# Supplementary material for: Health-related quality of life in young adults born small for gestational age: a prospective cohort study
Source: Health Qual Life Outcomes. 2022 Mar 24;20:49. doi: 10.1186/s12955-022-01948-4 (PMC8944049; doi:10.1186/s12955-022-01948-4)
Supplement: Supplementary file 2 — Additional file 2: Table A2 Health-related quality of life in participants born SGA at term and controls at 20 years [file 12955_2022_1948_MOESM2_ESM.docx]

**Table A2** Health-related quality of life in participants born SGA at term and controls at 20 years

|  | **SGA (n = 55)** | |  | **Control (n = 74)** | |  |  |  |  |
| --- | --- | --- | --- | --- | --- | --- | --- | --- | --- |
|  | **Mean** | **(SD)** |  | **Mean** | **(SD)** |  | **Mean difference (95% CI)^a^** | | ***p*-value** |
| *Domains* |  |  |  |  |  |  |  |  |  |
| Physical functioning | 95.3 | (7.1) |  | 95.6 | (10.0) |  | -0.2 | (-3.0 to 3.0) | 0.873 |
| Role-physical | 83.2 | (30.1) |  | 91.2 | (22.1) |  | -8.0 | (-17.6 to 0.9) | 0.108 |
| Bodily pain | 80.5 | (23.5) |  | 80.2 | (22.5) |  | 0.2 | (-8.1 to 8.4) | 0.957 |
| General health^b^ | 73.4 | (24.2) |  | 78.7 | (19.8) |  | -5.4 | (-13.3 to 2.2) | 0.169 |
| Vitality^b^ | 50.2 | (21.6) |  | 56.2 | (14.2) |  | -6.2 | (-12.7 to -0.3) | 0.063 |
| Social functioning | 81.6 | (22.7) |  | 92.7 | (13.1) |  | -11.3 | (-17.7 to -4.7) | 0.002 |
| Role-emotional | 70.3 | (38.8) |  | 90.5 | (23.7) |  | -20.4 | (-32.1 to -9.1) | 0.003 |
| Mental health^b^ | 70.0 | (18.1) |  | 79.2 | (11.9) |  | -9.4 | (-15.0 to -4.3) | 0.001 |
| *Component summaries* |  |  |  |  |  |  |  |  |  |
| Physical component summary^b^ | 55.9 | (7.3) |  | 54.9 | (6.1) |  | 0.9 | (-1.5 to 3.3) | 0.450 |
| Mental component summary^b^ | 44.0 | (11.9) |  | 51.0 | (7.7) |  | -7.1 | (-10.8 to -3.7) | <0.001 |

Domain scores are given in percentage (range 0-100) and higher scores indicate better health-related quality of life

Component summaries are given as T-scores based on an average of 50 points and a standard deviation of 10 points

CI, confidence interval; SD, standard deviation; SGA, small for gestational age

^a^ Mean difference adjusted for sex, confidence interval and *p*-value based on bias-corrected and accelerated bootstrap (BCa)

^b^ Data missing for one control participant
