# Supplementary material for: Health-related quality of life in young adults born small for gestational age: a prospective cohort study
Source: Health Qual Life Outcomes. 2022 Mar 24;20:49. doi: 10.1186/s12955-022-01948-4 (PMC8944049; doi:10.1186/s12955-022-01948-4)
Supplement: Supplementary file 3 — Additional file 3: Table A3 Health-related quality of life in participants born SGA at term and controls at 28 years [file 12955_2022_1948_MOESM3_ESM.docx]

**Table A3** Health-related quality of life in participants born SGA at term and controls at 28 years

|  | **SGA (n = 66)** | |  | **Control (n = 86)** | |  |  |  |  |
| --- | --- | --- | --- | --- | --- | --- | --- | --- | --- |
|  | **Mean** | **(SD)** |  | **Mean** | **(SD)** |  | **Mean difference (95% CI)^a^** | | ***p*-value** |
| *Domains* |  |  |  |  |  |  |  |  |  |
| Physical functioning | 94.2 | (12.7) |  | 96.1 | (7.6) |  | -2.0 | (-6.3 to 1.6) | 0.273 |
| Role-physical | 80.7 | (36.1) |  | 89.8 | (26.4) |  | -10.2 | (-21.0 to 0.5) | 0.050 |
| Bodily pain | 75.1 | (24.6) |  | 79.6 | (21.8) |  | -4.9 | (-13.0 to 2.4) | 0.203 |
| General health | 79.6 | (20.9) |  | 81.7 | (16.0) |  | -2.3 | (-9.2 to 4.0) | 0.459 |
| Vitality | 55.7 | (21.3) |  | 56.5 | (17.9) |  | -1.3 | (-7.5 to 4.7) | 0.687 |
| Social functioning | 85.4 | (23.8) |  | 93.2 | (15.2) |  | -7.9 | (-15.4 to -1.2) | 0.027 |
| Role-emotional | 88.9 | (24.3) |  | 92.6 | (21.3) |  | -3.8 | (-11.5 to 3.5) | 0.323 |
| Mental health | 78.4 | (16.3) |  | 80.8 | (13.4) |  | -2.6 | (-8.0 to 2.1) | 0.322 |
| *Component summaries* |  |  |  |  |  |  |  |  |  |
| Physical component summary | 53.3 | (8.9) |  | 54.8 | (6.7) |  | -1.7 | (-4.6 to 0.9) | 0.184 |
| Mental component summary | 50.3 | (9.2) |  | 51.8 | (8.4) |  | -1.5 | (-4.6 to 1.3) | 0.314 |

Domain scores are given in percentage (range 0-100) and higher scores indicate better health-related quality of life

Component summaries are given as T-scores based on an average of 50 points and a standard deviation of 10 points

CI, confidence interval; SD, standard deviation; SGA, small for gestational age

^a^ Mean difference adjusted for sex, confidence interval and *p*-value based on bias-corrected and accelerated bootstrap (BCa)
